# Nucleotide‐binding domain and leucine‐rich‐repeat‐containing protein X1 deficiency induces nicotinamide adenine dinucleotide decline, mechanistic target of rapamycin activation, and cellular senescence and accelerates aging lung‐like changes

**DOI:** 10.1111/acel.13410

**Published:** 2021-06-04

**Authors:** Hyeon Jun Shin, Sang‐Hun Kim, Hong‐Jai Park, Min‐Sun Shin, Insoo Kang, Min‐Jong Kang

**Affiliations:** ^1^ Section of Pulmonary, Critical Care and Sleep Medicine Department of Internal Medicine Yale University School of Medicine New Haven CT USA; ^2^ Section of Rheumatology, Allergy and Immunology Department of Internal Medicine Yale University School of Medicine New Haven CT USA

**Keywords:** cellular senescence, lung aging, mTOR (mechanistic target of rapamycin), NAD^+^ (nicotinamide adenine dinucleotide), NLRX1 (nucleotide‐binding domain and leucine‐rich‐repeat‐containing protein X1)

## Abstract

Mitochondrial dysfunction has long been implicated to have a causative role in organismal aging. A mitochondrial molecule, nucleotide‐binding domain and leucine‐rich‐repeat‐containing protein X1 (NLRX1), represents the only NLR family member that targets this cellular location, implying that NLRX1 probably establishes a fundamental link between mitochondrial functions and cellular physiology. However, the significance of NLRX1 function in cellular senescence, a key conceptual constituent in aging biology, is yet to be defined. Here, we demonstrate that molecular hallmarks involved in aging biology including NAD^+^ decline, and activation of mTOR, p53, and p16^INK4A^ are significantly enhanced in NLRX1 deficiency in vitro. Mechanistic studies of replicative cellular senescence in the presence or absence of NLRX1 in vitro reveal that NLRX1‐deficient fibroblasts fail to maintain optimal NAD^+^/NADH ratio, which instigates the decline of SIRT1 and the activation of mTOR, p16^INK4A^, and p53, leading to the increase in senescence‐associated beta‐galactosidase (SA‐β‐gal)‐positive cells. Importantly, the enhanced cellular senescence response in NLRX1 deficiency is significantly attenuated by pharmacological inhibition of mTOR signaling in vitro. Finally, our in vivo murine studies reveal that NLRX1 decreases with age in murine lungs and NLRX1 deficiency in vivo accelerates pulmonary functional and structural changes that recapitulate the findings observed in human aging lungs. In conclusion, the current study provides evidence for NLRX1 as a crucial regulator of cellular senescence and in vivo lung aging.

## Graphical abstract

Nucleotide‐binding domain and leucine‐rich‐repeat‐containing protein X1 (NLRX1)‐deficient fibroblasts fail to maintain optimal NAD^+^/NADH ratio, which instigates the decline of SIRT1 and the activation of mTOR, p16INK4A, and p53, leading to the increase in senescence‐associated beta‐galactosidase‐positive cells. NLRX1 decreases with age in murine lungs, and NLRX1 deficiency in vivo accelerates aging lung‐like functional and structural changes. The current study provides evidence for NLRX1 as a crucial regulator of cellular senescence and in vivo lung aging.

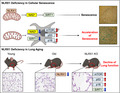

**Abbreviations**
mTOR: Mechanistic target of rapamycin
NAD^+^
: Nicotinamide Adenine Dinucleotide
NLRX1: nucleotide‐binding domain and leucine‐rich‐repeat‐containing protein X1
SA‐β‐gal: Senescence‐associated beta‐galactosidase
SIRT1: Sirtuin 1

## 1 INTRODUCTION

A decline in mitochondrial quality and activity has been associated with normal aging and correlated with the development of a wide range of age‐related diseases (Sun et al., 2016). Multiple lines of evidence from literature support mitochondria take important parts not only in cellular respiration but also in fundamental cellular functions including metabolism, innate immune signaling, and regulated cell death responses. Consequently, mitochondrial dysfunction can be mechanically linked to aging and age‐related diseases through cellular senescence, stem cell exhaustion, inflammaging, and other pathobiological processes (Kang & Shadel, 2016; Sun et al., 2016).

A mitochondrial molecule, nucleotide‐binding domain and leucine‐rich‐repeat‐containing protein X1 (NLRX1), is a member of the NLR family of pattern recognition receptors (PRRs) that has a unique N‐terminal domain, which accounts for the letter “X” in its acronym(Moore et al., 2008). It contains a mitochondrial targeting sequence, and biochemical analyses demonstrate that NLRX1 localizes to the mitochondria (Arnoult et al., 2009; Moore et al., 2008). Strikingly, NLRX1 represents the first, and so far only, example of a PRR family member that targets this cellular location, implying that NLRX1 probably establishes a fundamental link between mitochondrial functions and cellular physiology (Arnoult et al., 2011; Nagai‐Singer et al., 2019). NLRX1 has been shown to negatively regulate type‐I interferon, attenuate pro‐inflammatory NF‐κB signaling, and modulate autophagy, cell death, and proliferation (Nagai‐Singer et al., 2019). The role of NLRX1 in aging biology, however, has rarely been explored.

Cellular senescence is defined as a cell state triggered by stressful insults and certain physiological processes characterized by a prolonged and generally irreversible cell‐cycle arrest with secretory features, macromolecular damage, and altered metabolism (Gorgoulis et al., 2019). Cellular senescence has emerged as a potentially important contributor to aging and age‐related diseases, and it is an attractive target for therapeutic exploitation (Childs et al., 2015; van Deursen, 2019). Importantly, the significance of NLRX1 function in cellular senescence is yet to be defined.

The structural changes leading to a progressive decline in function of the pulmonary system with normative age, often designated as “aging lung” or “senile lung,” have been noted for a long time (Fukuchi, 2009; Lowery et al., 2013; Verbeken et al., 1992). The characteristics of aging lung include (a) increased diameter of alveolar ducts and alveoli with loss of alveolar surface area, leading to diminished gas exchange; (b) decreased capillary density, contributing to lower diffusion capacity and (c) changes in inflammatory processes and alteration in immune cell functions (Fukuchi, 2009; Lowery et al., 2013; Skloot, 2017). The genetic, molecular, and cellular mechanisms involved in lung aging, however, are still poorly understood (Budinger et al., 2017; Cho & Stout‐Delgado, 2020; Thannickal et al., 2015).

From our previous study that established the pathogenic role of NLRX1 in chronic obstructive pulmonary disease (COPD) for which aging is an important risk factor (Kang et al., 2015), we questioned what functional significance NLRX1 may have in cellular senescence and the biology of lung aging. Here, utilizing in vitro experiments of replicative senescence in fibroblasts and an in vivo murine model of normative aging, we demonstrate that NLRX1 deficiency enhances replicative senescence and accelerates pulmonary functional and structural changes that recapitulate the findings observed in human aging lungs, suggesting the age‐associated reduction in NLRX1 may play an important role in lung aging.

## 2 RESULTS

### 2.1 Replicative cellular senescence is enhanced in NLRX1 deficiency

**FIGURE 1:**
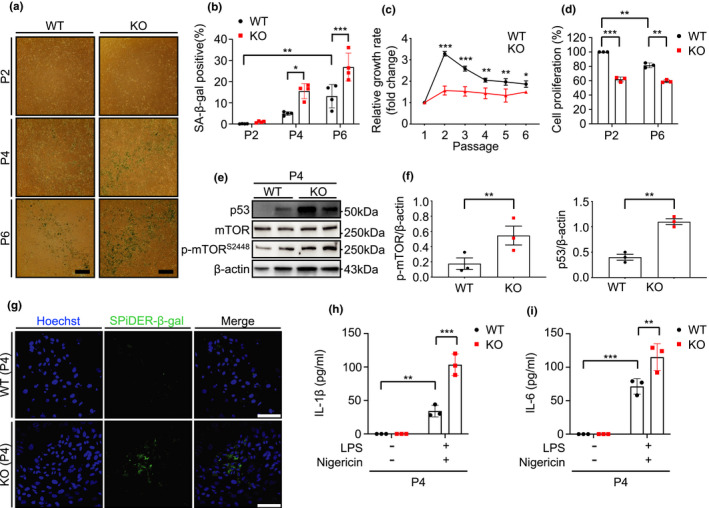
Nucleotide‐binding domain and leucine‐rich‐repeat‐containing protein X1 deficiency accelerates cellular senescence in mouse embryonic fibroblasts. Primary mouse embryonic fibroblasts (MEFs) from wild‐type (WT) or NLRX1 knockout (KO) mice were subcultured continuously for induction of replicative senescence. (a) Senescence‐associated beta‐galactosidase (SA‐β‐gal) staining to examine the population of senescent cells. Scale bars, 200 μm. (b) Graphs showing the percentage of SA‐β‐gal‐positive cell at each passage (P; n = 4). (c) Relative growth rate at each passage (n = 3). (d) MTT assay to investigate cell proliferation in passages 2 and 6 (n = 3). (e) Immunoblot and (f) Densitometry evaluation to detect p53, mTOR, and phosphorylation of mTOR at serine 2448 (p‐mTOR^S2448^) in WT or NLRX1 KO MEFs at passage 4. β‐actin used as loading control. (g) SA‐β‐gal fluorescence staining using SPiDER‐β‐gal to examine the population of senescent cells measured by confocal microscopy. Hoechst 33342 was used for nucleus staining. Scale bars, 50 μm. WT or NLRX1 KO MEFs at passage 4 were treated Nigericin for 1 h after primed LPS for 4 h for activation of inflammasome. (h) ELISA assay to detect IL‐1β and (i) IL‐6 secretion in supernatants from inflammasome‐induced WT or NLRX1 KO MEFs (n = 3). Error bars indicate means ± SD. Data were analyzed by two‐tailed unpaired t test (f) or two‐way ANOVA followed by Tukey's multiple comparisons test. ***p < 0.005, **p < 0.01, *p < 0.05

To define the role of NLRX1 in replicative cellular senescence in vitro, we chose mouse embryonic fibroblasts (MEFs) that are known to be suitable for senescence studies (Di Micco et al., 2008; Wright & Shay, 2000). When isolated MEFs from wild‐type (WT) or NLRX1 null mutant (KO) mice were subcultured in vitro continuously for the induction of replicative senescence, NLRX1‐deficient MEFs (MEF^KO^) showed increases in senescence‐associated beta‐galactosidase (SA‐β‐gal)‐positive (+) cells compared with those of WT MEFs (MEF^WT^). Specifically, in NLRX1 deficiency, the SA‐β‐gal (+) cell population was significantly increased in an earlier passage (Passage [P] 4) compared with those of WT MEFs (MEF^WT^), and the difference was more enhanced in a later passage (P6; Figure 1a,b). In line with this, evaluation of the relative growth ratio revealed that MEF^KO^ cells showed a significantly lower number in each passage compared with those of MEF^WT^ cells (Figure 1c). From MEF^KO^ cells, we also observed the reduction in cell proliferation rate in early‐ and late‐passage (Figure 1d). Of note, all experiments of replicative senescence were undertaken up to P6 because MEF^KO^ cells failed to proliferate further after P6 (data not shown); our observation is further supported by the literature demonstrated that MEFs proliferated until passage 5–6, after which time their population doubling time was dramatically increased (Manning & Kumar, 2010). Next, we evaluated molecular markers related to replicative senescence. As known, the tumor suppressor p53 is a well‐established inducer of cellular senescence (Vousden & Prives, 2009), and the activation of the mechanistic target of rapamycin (mTOR) is required for the induction of senescence (Demidenko et al., 2009). Indeed, we confirmed the increased expression of p53, phosphorylation of mTOR at serine 2448 (p‐mTOR^S2448^), and fluorescence‐stained SA‐β‐gal (SPiDER‐β‐gal) activity, respectively, from MEF^KO^ compared with MEF^WT^ cells at P4 (Figure 1e‐g). Furthermore, after stimulation with LPS and nigericin, MEF^KO^ cells showed a significant enhancement of active IL‐1β production and IL‐6 secretion, respectively, compared with MEF^WT^ cells (Figure 1h,i).

### 2.2 Nucleotide‐binding domain and leucine‐rich‐repeat‐containing protein X1 plays an inhibitory function in replicative senescence

**FIGURE 2:**
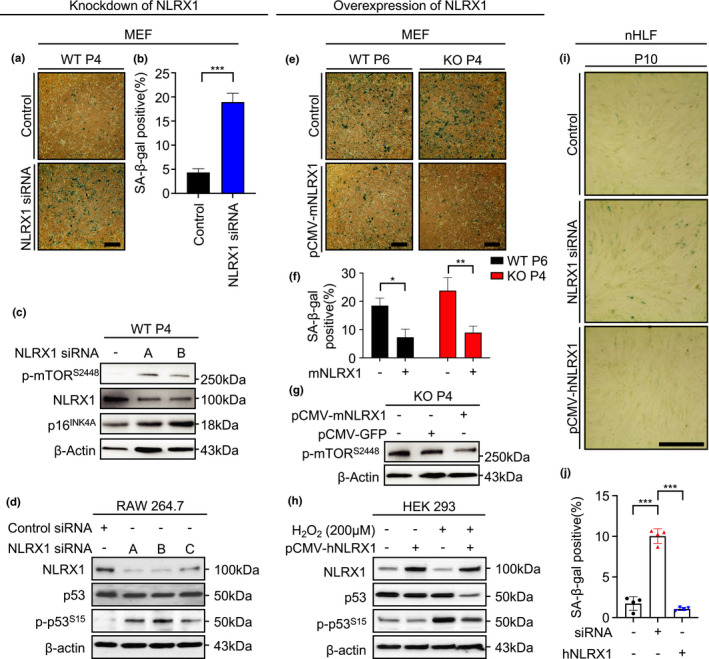
Nucleotide‐binding domain and leucine‐rich‐repeat‐containing protein X1 plays an inhibitory role in cellular senescence. (a‐c) WT MEFs transfected with NLRX1 silencing RNA (siRNA) for knockdown of NLRX1. (a) SA‐β‐gal staining to investigate the alteration of SA‐β‐gal‐positive cells in WT MEFs at passage (P) 4. Scale bars, 200 μm. (b) Graphs showing the percentage of SA‐β‐gal‐positive cells at passage 4 (n = 3). (c) Immunoblot to detect p‐mTOR^S2448^ and NLRX1 in WT MEFs. (d) RAW 264.7 cells were transfected with control silencing RNA (siRNA) or three types of NLRX1 siRNA (A, B, and C). Then, immunoblot to examine expression levels of NLRX1, p53, and p‐p53^S15^ in whole‐cell lysates. (e‐g) P6 WT or P4 NLRX1 KO MEFs transfected with an NLRX1 expression vector (pCMV‐mNLRX1) for overexpression of NLRX1. (e) SA‐β‐gal staining to investigate alteration of SA‐β‐gal‐positive cells. Scale bars, 200 μm. (f) Graphs showing the percentage of SA‐β‐gal‐positive cell (n = 3). (g) Immunoblot to detect p‐mTOR^S2448^. (h) HEK 293 cells were transfected with pCMV‐GFP or pCMV‐hNLRX1. Then, immunoblot to examine expression levels of NLRX1, p53, and p‐p53^S15^ in whole‐cell lysates. pCMV‐GFP used as the control of pCMV‐hNLRX1. β‐actin used as loading control. (i) SA‐β‐gal staining to investigate the alteration of SA‐β‐gal‐positive cells after transfection of NLRX1 siRNA or pCMV‐hNLRX1 in normal human lung fibroblasts (nHLF) cells. Scale bars, 200 μm. (f) Graphs showing the percentage of SA‐β‐gal‐positive cell (n = 4). Error bars indicate means ± SD. Data were analyzed by two‐tailed unpaired t test (b) or one‐way (j) or two‐way (f) ANOVA followed by Tukey's multiple comparisons test. ***p < 0.005, **p < 0.01, *p < 0.05

To prove the causative relation of NLRX1 with cellular senescence or mTOR and p53 activation, we proceeded with NLRX1‐knockdown or NLRX1 overexpression experiments using silencing RNA (siRNA) or expression plasmid of NLRX1 (pCMV‐mNLRX1 or pCMV‐hNLRX1). Indeed, NLRX1 siRNA‐transfected MEF^WT^ showed a further increase in SA‐β‐gal (+) cells and elevation of p‐mTOR^S2448^ and p16^INK4A^ expressions (Figure 2a‐c). Additionally, NLRX1‐knockdown experiments demonstrated a significant increase in the phosphorylation of p53 at serine 15 (p‐p53^S15^) expression with all three different types of NLRX1 siRNA in mouse macrophage‐like cell (RAW 264.7; Figure 2d). In contrast, markers of cellular senescence were significantly decreased after NLRX1 overexpression. Specifically, P6 MEF^WT^ and P4 MEF^KO^ cells, respectively, showed a significant decrease in SA‐β‐gal (+) cells after pCMV‐mNLRX1 transfection (Figure 2e,f), and pCMV‐mNLRX1 transfected MEF^KO^ showed a significant reduction in p‐mTOR^S2448^ expression level (Figure 2g). Additionally, when human embryonic kidney cells (HEK293) were treated with hydrogen peroxide (H_2_O_2_) to induce oxidative stress‐induced premature senescence, NLRX1‐overexpressing HEK293 cells showed a significant attenuation of p53 and p‐p53^S15^ expression (Figure 2h). Importantly, the negative regulation of SA‐β‐gal (+) cells via an NLRX1‐dependent manner was also observed from normal human lung fibroblasts (nHLF; Figure 2i,j).

### 2.3 Nucleotide‐binding domain and leucine‐rich‐repeat‐containing protein X1 deficiency causes NAD^+^ decline in vitro and in vivo

**FIGURE 3:**
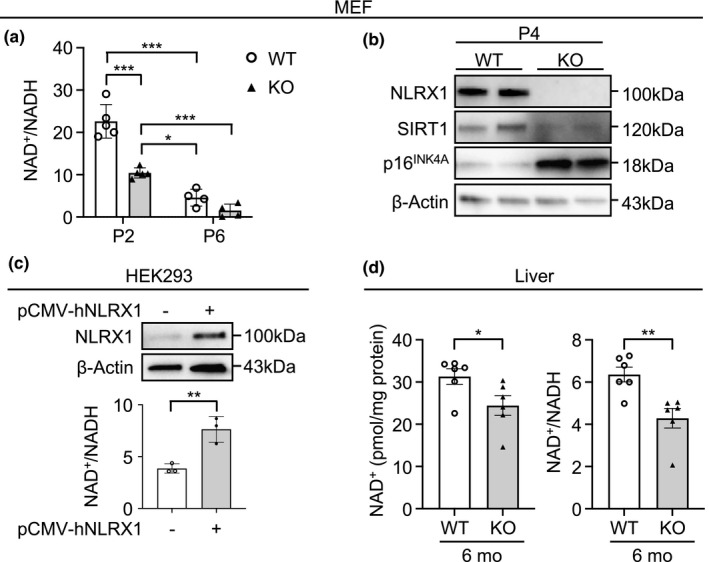
Nucleotide‐binding domain and leucine‐rich‐repeat‐containing protein X1 deficiency causes NAD^+^/NADH imbalance in vitro and in vivo. (a) Colorimetric assay to examine NAD^+^/NADH ratio in WT or NLRX1 KO mouse embryonic fibroblasts (MEFs) at passage 2 or 6. n = 5 at passage 2, n = 4 at passage 6. (b) Immunoblot to determine expression levels of SIRT1, NLRX1, and p16^INK4A^ in WT or NLRX1 KO MEFs at passage 4. (c) HEK293 cells were transfected with a human NLRX1 expression vector (pCMV‐hNLRX1) for upregulation of NLRX1 expression. Immunoblot to detect NLRX1 expression level (upper panel). Colorimetric assay to examine NAD^+^/NADH ratio in NLRX1‐overexpressed HEK293 cells (bottom panel; n = 3). (d) Colorimetric assay to examine NAD^+^ level (left panel) or NAD^+^/NADH ratio (right panel) in 6‐months liver from WT or NLRX1 KO mice (n = 6, each group). Error bars indicate means ± SD (a and c) or SEM (d). Data were analyzed by two‐tailed unpaired t test (c and d) or two‐way ANOVA followed by Tukey's multiple comparisons test (a). ***p < 0.005, **p < 0.01, *p < 0.05

To further explore the functional significance and the underlying mechanism by which cellular senescence and its molecular markers are enhanced in NLRX1 deficiency, we questioned whether NLRX1 may play a role in nicotinamide adenine dinucleotide (NAD^+^) homeostasis, a currently highlighted topic in aging biology (Katsyuba et al., 2020). Indeed, replicative senescent‐MEFs showed a significant reduction in NAD^+^/NADH ratio at P6 compared with P2 (Figure 3a), and, interestingly, a significant reduction in the NAD^+^/NADH ratio was observed from MEF^KO^ cells compared with MEF^WT^ cells at an earlier passage (P2) and a later passage (P6; Figure 3a). As known, silent information regulator 2 (Sir2) family (sirtuins)‐1 (SIRT1) protein is an NAD^+^‐dependent deacetylase that plays a critical role in aging and age‐associated pathologies (Hall et al., 2013). Thus, when the molecular alteration of SIRT1 was evaluated in the presence or absence of NLRX1, a significant decrease in SIRT1 expression was observed from MEF^KO^ cells compared with MEF^WT^ cells (Figure 3b). In line with this, a significant increase in p16^INK4A^ expression was observed from MEF^KO^ cells compared with MEF^WT^ cells (Figure 3b). Moreover, our additional NLRX1‐overexpression experiments with HEK293 cells indicated a causative link of NLRX1 deficiency to NAD^+^ decrease in cells, by demonstrating that NLRX1‐overexpressed HEK293 cells showed a significant increase in NAD^+^/NADH ratio (Figure 3c).

To investigate whether NLRX1 deficiency may lead to NAD^+^ decline in vivo, we measured NAD^+^ level and NAD^+^/NADH ratio from the liver. As known, the liver and, to a lesser extent, the kidney appear to act as a hub for whole‐body NAD^+^ homeostasis; all enzymes involved in NAD^+^ biosynthesis, with only few exceptions, are highly expressed in these two organs (Katsyuba et al., 2020). Indeed, in line with our in vitro experiments, 6‐month‐old (mo) NLRX1 KO mice revealed significantly decreased levels of both NAD^+^ and NAD^+^/NADH ratio (Figure 3d).

### 2.4 Pharmacological mTOR inhibition restores NLRX1 deficiency‐induced enhance senescence

**FIGURE 4:**
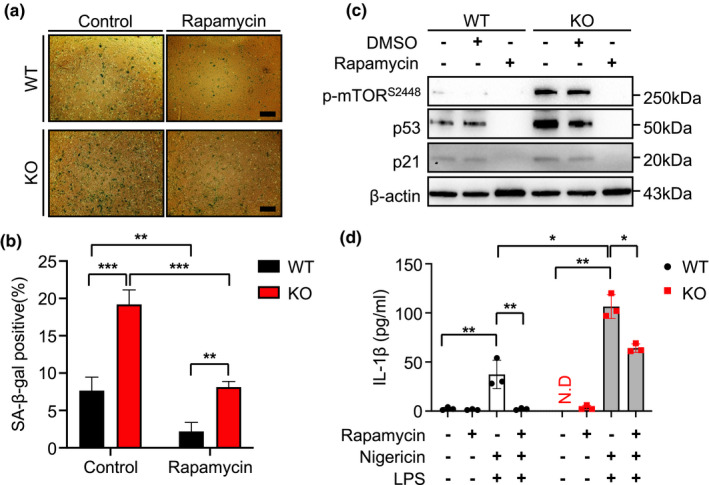
Inhibition of mTOR attenuates cellular senescence in mouse embryonic fibroblasts. (a) WT or NLRX1 KO mouse embryonic fibroblasts (MEFs) at passage (P) 4 were SA‐β‐gal stained after rapamycin (100 nM, 16 h) treatment. Scale bars, 200 μm. (b) Graphs showing the percentage of SA‐β‐gal‐positive cell (n = 3). (c) Immunoblot to detect expression levels of p‐mTOR^S2448^, p53, and p21 in WT or NLRX1 KO at P4 after rapamycin (100 nM, 16 h) treatment. β‐actin used as a loading control. (d) ELISA assay to detect IL‐1β in WT or NLRX1 KO MEFs at P4 were pre‐treated rapamycin (100 nM, 16 h) before LPS (1 μg/ml, 4 h)/Nigericin (5 μM, 1 h) treatment (n = 3). Error bars indicate means ± SD. Data were analyzed by two‐way ANOVA followed by Tukey's multiple comparisons test. ***p < 0.005, **p < 0.01, *p < 0.05. N.D. means not detected

Next, we evaluated whether pharmacologic inhibition of mTOR signaling may ameliorate the enhanced cellular senescence observed from NLRX1‐deficiency cells. Specifically, MEFs in the presence or absence of NLRX1 at P 4 were treated with rapamycin (100 nM) for 16 h, and SA‐β‐gal activity, molecular alterations with cellular senescence, and IL‐1β production after stimulation with LPS and nigericin, respectively, were evaluated and compared. As expected from the literature, rapamycin‐treated MEF^WT^ cells showed significantly decreased levels of SA‐β‐gal (+) cells (Figure 4a,b), molecular expression of p53 and p21 (Figure 4c), and LPS/Nigericin‐induced IL‐1β production (Figure 4d). Importantly, these enhanced responses of SA‐β‐gal (+) cells, molecular expression of p53 and p21, and LPS/Nigericin‐induced IL‐1β production, respectively, that are observed in NLRX1 deficiency, were significantly ameliorated with the rapamycin treatment (Figure 4a‐d). Taken together, these results suggest that the NLRX1‐NAD^+^‐mTOR axis may play a causative role in the enhanced cellular senescence observed in NLRX1 deficiency.

### 2.5 Nucleotide‐binding domain and leucine‐rich‐repeat‐containing protein X1 decreases with normative aging and aging lung‐like changes are accelerated in NLRX1 deficiency in vivo

**FIGURE 5:**
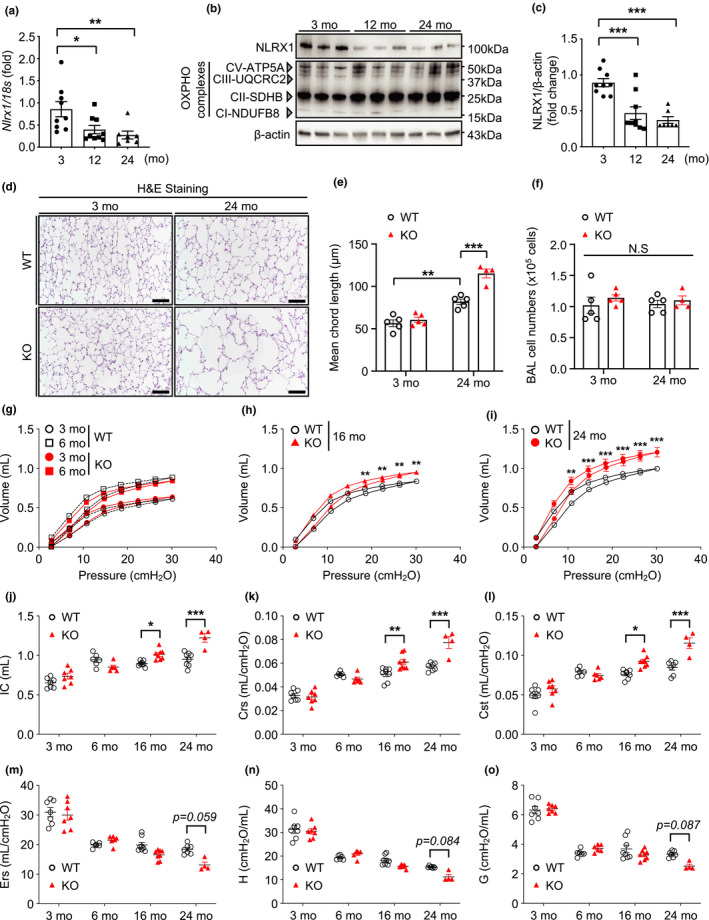
Nucleotide‐binding domain and leucine‐rich‐repeat‐containing protein X1 decreases with age and NLRX1 deficiency accelerates aging lung‐like structural and functional changes. (a) Real‐time PCR to detect the expression levels of *Nlrx1* mRNA in lungs of 3‐ or 12‐ or 24‐month‐old (mo) wild‐type (WT) mice. (b) Immunoblot to detect NLRX1 in lungs of 3‐ or 12‐ or 24‐months WT mice. (c) Densitometry evaluation of the NLRX1 protein expression levels (3‐months, n = 9; 12‐months, n = 9; 24‐months, n = 7). (d) Representative images of H&E‐stained lung sections from WT or NLRX1 knockout (KO) mice. Scale bars, 200 μm. (e) Quantification of mean chord length (μm) is shown in the graphs, and (f) Total cell recovery of bronchoalveolar lavage (BAL) fluids from WT or NLRX1 KO mice. Each group n = 5 mice except 24‐months NLRX1 KO (n = 4). For (g–o), pulmonary function tests from 3‐ or 6‐ or 16‐ or 24‐months WT or NLRX1 KO mice were measured by flexiVent system. WT or NLRX1 KO 3‐months, n = 7; WT or NLRX1 KO 6‐months, n = 6; WT or NLRX1 KO 16‐months, n = 8; WT 24‐months, n = 7; NLRX1 KO 24‐months, n = 4. * Note: Two of six NLRX1 KO mice allocated for the 24‐months group succumbed to natural death during the follow‐up period. (g–i) Pressure‐volume loop at indicated timepoints. (j) Inspiratory capacity (IC), (k) Dynamic compliance of respiratory system (Crs), (l) Static compliance of respiratory system (Cst), (m) elastance of respiratory system (Ers), (n) tissue elastance (H), and (o) tissue damping (g). * Note: Significance included in the graph indicated only the difference between the WT and KO groups. Refer to Figure S2 for the difference in significance within each group. Error bars indicate means ± SEM. Data were analyzed by ordinary one‐way ANOVA followed by Tukey's multiple comparisons test (a and c), or two‐way ANOVA followed by Tukey's multiple comparisons test (e–o). ***p < 0.005, **p < 0.01, *p < 0.05. N.S. means not significant

Our previous study reported the pathogenic role of NLRX1, which may contribute to the development of chronic obstructive pulmonary disease (COPD) (Kang et al., 2015). Additionally, there is growing evidence that aging hallmarks are noticeable features of COPD (Easter et al., 2020; Kang, 2020). Indeed, when we assessed the level of NLRX1 expression with normative aging in murine lungs, both mRNA and protein expression levels of NLRX1 were significantly reduced in 12‐ and 24‐month‐old (mo) mice compared with those of young (3‐months) mice (Figure 5a‐c and Figure S1a,b).

To investigate the role of NLRX1 in aging‐associated pulmonary structural and functional alterations, we proceeded with lung morphometric evaluation and pulmonary function tests from WT or NLRX1 KO mice with normative aging. The histology of 24‐months WT murine lungs showed modest enlargement of alveolar space which resembles human aging lung and, interestingly, the enlarged alveolar space was significantly enhanced in the lungs of 24‐months NLRX1 KO mice (Figure 5d and Figure S1c,d). Lung morphometric evaluation of mean chord length confirmed this observation (Figure 5e). Total cell recovery from bronchoalveolar lavage (BAL) fluids, which is an indicator often increased with various lung pathologies, was not different in the presence or absence of NLRX1 (Figure 5f). In accord with the structural changes noted above, WT mouse lungs demonstrated pulmonary functional alterations which resemble human aging lung with normative aging, and, importantly, these functional alterations were accentuated in NLRX1 deficiency in vivo (Figure 5g‐o and Figure S2a‐f). Specifically, compared with the WT group, the NLRX1 KO group showed significantly enhanced pressure‐volume loop (PV loop), inspiratory capacity (IC), dynamic compliance of respiratory system (Crs), and static compliance of respiratory system (Cst) at the 16‐ or 24‐month time point, respectively (Figure 5g‐l). Additionally, 24‐months NLRX1 KO mice presented decreasing patterns elastance of respiratory system (Ers; *p* = 0.059), tissue elastance (H; *p* = 0.084), and tissue damping (G; *p* = 0.087) compared with those of WT mice (Figure 5m‐o). Furthermore, as the mice grew older, the age‐dependent alterations of the above lung function variables were more markedly exaggerated in NLRX1 KO mice compared with WT mice (Figure S2a‐f). Of note, two of six NLRX1 KO mice allocated for the 24‐months group succumbed to natural death during the follow‐up period.

### 2.6 Mechanistic target of rapamycin signaling and p53 with p16^INK4A^ are activated in NLRX1‐deficient lungs in vivo

**FIGURE 6:**
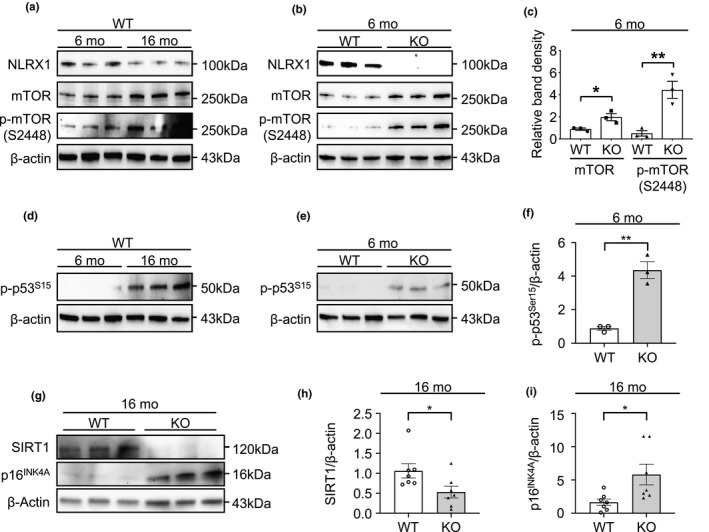
Nucleotide‐binding domain and leucine‐rich‐repeat‐containing protein X1‐dependent alterations of mTOR, p53, and p16^INK4A^ in murine lungs. (a) Immunoblot to detect NLRX1, mTOR, and phosphorylation of mTOR at serine 2448 (p‐mTOR^S2448^) in lungs from 6‐ or 16‐month‐old (mo) wild‐type (WT) mice. (b) Immunoblot to detect NLRX1, mTOR, and p‐mTOR^S2448^ in lungs from 6‐months WT or NLRX1 knockout (KO) mice. (c) Densitometry evaluation of the relative mTOR or p‐mTOR^S2448^ protein expression levels. n = 3 mice, each group. (d) Immunoblot to evaluate the phosphorylation status of p53 at serine 15 (p‐p53^S15^) in lungs of 6‐ or 16‐months WT and (e) 6‐months WT or NLRX1 KO mice. (f) Densitometry evaluation of the relative p‐p53^S15^ protein expression levels. n = 3 mice, each group. (g) Immunoblot to detect SIRT1 and p16^INK4A^ in lungs from 16‐month‐old (mo) wild‐type (WT) or NLRX1 knockout (KO) mice. (h) Graph showing the relative SIRT1 and (i) p16^INK4A^ protein expression levels. n = 7 mice, each group. β‐actin used as a loading control. Error bars indicate means ± SEM. Data were analyzed by a two‐tailed unpaired t test. ***p < 0.005, **p < 0.01, *p < 0.05

We also investigated whether the molecular markers observed in the above in vitro studies are also altered via an NLRX1‐dependent manner in our normative lung aging model. As expected, the expression levels of mTOR and p‐mTOR^S2448^ were increased with normative aging in lungs from WT mice, and, indeed, these molecular alterations were significantly accentuated in NLRX1 deficiency in vivo (Figure 6a). Interestingly, NLRX1 KO mice showed a modest increase in mTOR activation at a 6‐months time point, indicating that molecular markers associated with aging are activated from an earlier age in NLRX1 deficiency (Figure 6b,c). Of note, no difference in mTOR activation status at 1‐month or 2‐months time point, respectively, was noted in the presence or absence of NLRX1, suggesting that accelerated aging in NLRX1 deficiency in vivo is unlikely attributed to developmental causes (Figure S3a,b. Likewise, the expression level of p‐p53^S15^ was increased in lungs from 16‐months mice (Figure 6d) and was significantly enhanced in NLRX1 deficiency (Figure 6e,f). Finally, in line with the above findings, with normative aging, lungs from NLRX1 KO mice showed a significant decline of SIRT1 protein and a significant increase in p16^INK4A^ protein, respectively, compared with WT controls (Figure 6g‐i).

## 3 DISCUSSION

The current study reveals multiple novel findings, which provide an enhanced mechanistic insight for the biology of lung aging; these comprise (a) the alterations of multiple molecular hallmarks of cellular senescence in NLRX1‐deficient fibroblasts; (b) the significance of the NLRX1‐NAD^+^‐mTOR axis in cellular senescence; (c) the reduction in NLRX1 in murine lungs with normative aging; and (d) the enhanced structural and functional alterations in NLRX1 deficiency in vivo, which resemble human aging lung.

Since initially identified as a negative regulator of mitochondrial antiviral signaling (MAVS) protein, the functional significance of NLRX1 in biology and medicine has immensely expanded, revealing a crucial role of NLRX1 in a multitude of animal disease models in vivo, including cancer, virus infection, osteoarthritis, traumatic brain injury, inflammatory bowel disease, autoimmune encephalomyelitis, and COPD (reviewed in reference [Nagai‐Singer et al., 2019]). However, our understanding of how NLRX1 functions in cellular senescence and aging biology is still limited. By demonstrating that key molecular events currently highlighted in aging biology such as NAD^+^, SIRT1, and mTOR are significantly altered via an NLRX1‐dependent manner, the current study adds important knowledge to aging biology.

Over the last decade, the actions of NAD^+^ have been markedly extended from being an oxidoreductase cofactor for enzymatic activities to acting as a substrate for a wide range of proteins including NAD^+^‐dependent protein deacetylases (e.g., sirtuins), poly(ADP‐ribose) polymerases, and transcription factors that affect a large array of cellular functions (Houtkooper et al., 2010). For example, SIRT1, a famous anti‐aging molecule, is an NAD^+^‐dependent deacetylase with the capacity to negatively regulate mTOR activation (Ghosh et al., 2010; Grabowska et al., 2017). There is growing evidence that NAD levels decline during chronological aging and that this decline is both a consequence of the aging process and also a contributor to the development of age‐related cellular dysfunction (Chini et al., 2017; Fang et al., 2017). Additionally, senescence phenotypes and mitochondrial dysfunction that are implicated in aging and in premature aging diseases such as ataxia telangiectasia are critically dependent on NAD^+^ (Fang et al., 2014; Yang et al., 2021). Intriguingly, our data revealed that the levels of NAD^+^ and NAD^+^/NADH ratio were significantly decreased in NLRX1 deficiency in vitro and in vivo, and our in vitro experiments implicate a causative link of endogenous NLRX1 deficiency to the decrease in NAD^+^ in cells. Collectively, NLRX1 may play an intrinsic role in homeostatic maintenance of NAD^+^/NADH in cells and, in NLRX1 deficiency, the failure of optimal NAD^+^/NADH maintenance may cause accelerated cellular senescence and contribute to the pathobiology of lung aging.

The mTOR molecule is highlighted as a key modulator of aging in evolutionarily divergent organisms, ranging from yeast to rodents, and this function has likely been conserved to some extent in humans (Johnson et al., 2013; Saxton & Sabatini, 2017). Multiple studies showed that rapamycin, a known mTOR inhibitor, extended lifespan in yeast, nematodes, fruit flies, and mice, firmly establishing mTOR signaling as a central, evolutionarily conserved regulator of longevity (Johnson et al., 2013). Indeed, the accelerated cellular senescence responses observed in NLRX1 deficiency were markedly ameliorated with rapamycin treatment. Our observations are supported by other studies. NLRX1‐deficient intestinal organoids showed lower levels of NAD^+^ and the expression of *Sirt1* was decreased (Leber et al., 2018); and SIRT1, an NAD^+^‐dependent deacetylase, can negatively regulate mTOR signaling in a TSC2‐dependent manner and is considered to play a role in protecting and regulating cellular fates from p53‐dependent senescence and aging (Ghosh et al., 2010; Ong & Ramasamy, 2018). Taken together, NLRX1 may have an important inhibitory function to limit cellular senescence and, in NLRX1 deficiency, reduced NAD^+^ level may lower SIRT1 activity, leading to the activation of senescence triggers such as mTOR.

It is a major challenge to elucidate how scientific advances in molecular insights of aging biology can be integrated into a conceptual framework that will illuminate the mechanistic understanding of lung aging and its related disorders (Budinger et al., 2017). Besides, the body of scientific knowledge related to lung aging and its contribution to the pathobiology of chronic lung diseases is lagging far behind other recent advances in aging biology (Kang, 2020). Regarding this, we previously reported that, from three independent human COPD cohorts, the expression of NLRX1 was suppressed in the lungs from patients with COPD and this suppression showed a strong correlation with the degree of airflow limitation, a hallmark of COPD (Kang et al., 2015). By revealing that NLRX1 declines in murine lungs with normative aging and its deficiency entails key molecular features highlighted in aging biology, the current study might throw light on a perspective that NLRX1 may be an important link molecule through which molecular understanding obtained from aging studies can be integrated for an enhanced mechanistic explanation of lung aging‐related disorders.

The current study brings several intriguing questions in the related research field. First, given that much attention is recently being paid to NAD^+^ biology in aging studies, a curiosity is raised about how endogenous NLRX1 deficiency fails to maintain optimal NAD^+^/NADH homeostasis in cells and in organs in vivo. For NAD^+^/NADH homeostasis depends on several variables, including the cellular redox state and the rates of NAD^+^ synthesis and NAD^+^ consumption (Verdin, 2015), comprehensive investigations will be required to elucidate each aspect further to gain a better understanding of its underlying mechanism(s). Second, further studies of mechanistic explanations should be pursued about how the reduction in NLRX1 may occur with normative aging in murine lungs in vivo, leading to the aging lung‐like accelerated structural and functional alterations. Third, recent studies suggest that NAD^+^ is central to mitochondrial homeostasis, including in mitochondrial biogenesis, mitophagy, the mitochondrial unfolded protein response (UPRmt), and nuclear‐mitochondrial communication (Fang et al., 2014; Gomes et al., 2013; Lautrup et al., 2019; Mouchiroud et al., 2013). These observations might provide important mechanistic clues for our findings; specifically, the impairment of mitochondrial homeostasis caused by the failure of optimal NAD^+^/NADH homeostasis, which is directly or indirectly related to NAD^+^‐dependent mitophagy, might function as a driving force of accelerated lung aging in NLRX1 deficiency in vivo. Fourth, our observations may provide molecular insights into the underlying mechanisms of human aging lung. Further translational or clinical studies might be fruitful to define whether a similar reduction in NLRX1 is observed in humans and, as noted in the current study, NLRX1 decline‐NAD^+^ decline‐cellular senescence has a functional significance in human aging lungs. Last but not least, it will be interesting to test the possibility of modulating NLRX1 expression or function as a novel approach for improving lung health or lung aging‐related diseases.

In conclusion, our study provides evidence that NLRX1 plays as a crucial regulator of cellular senescence and offers an in vivo murine model system that can be useful to elucidate the intersection of aging biology and the pathobiology of lung diseases.

## 4 EXPERIMENTAL PROCEDURES

### 4.1 Animals

Wild‐type (WT; from Jackson Laboratories) or NLRX1 knockout (KO; from Dr. J. P. Ting, University of North Carolina) mice were all on a C57BL/6J background and bred at Yale University. All animal experiments were approved by the Yale Animal Care and Use Committee (YACUC). All aged mice were obtained from housing the mice in the same environment under normative aging.

### 4.2 Cell isolation and culture

Mouse embryonic fibroblasts (MEFs) were generated from embryonic day 13.5 (E13.5) embryos of wild‐type (WT) and NLRX1 knockout (KO) mice following standard procedures, as described previously (Jozefczuk et al., 2012). MEFs were cultured in vitro in a humidified atmosphere of 5% CO_2_ at 37°C in Dulbecco's modified Eagle's medium (DMEM, Gibco) supplemented with 10% fetal bovine serum (FBS, Gibco), penicillin (50 IU/ml), and streptomycin (50 mg/ml, Gibco). All the experiments using MEFs were done at passage 2–6.

Human embryonic kidney (HEK) 293 cells (ATCC) were cultured in minimum essential media (MEM; Gibco) and incubated in 5% CO_2_ at 37°C. The medium was supplemented with 10% FBS, penicillin, and streptomycin (Gibco). Normal human lung fibroblasts (nHLFs) purchased from ATCC were cultured in Fibroblast basal medium with fibroblast growth kit (Low serum, ATCC). The medium was supplemented with 10% FBS, penicillin, and streptomycin (Gibco). For bronchoalveolar lavage (BAL) collection, the trachea was cannulated and lavaged two times with 0.9 ml PBS. Samples were centrifuged at 200*g* for 5 min, and the cell pellets were recovered in 200 μl sterile PBS, and total cell count of BAL was done using automated cell count using a Coulter Analyzer (Beckman Coulter).

### 4.3 Transfection and reagents

For regulation of NLRX1 expression level, MEFs or RAW264.7 or nHLF were transfected with Control siRNA or NLRX1 siRNA or pCMV‐GFP plasmid or pCMV‐mNLRX1 or pCMV‐hNLRX1 plasmid (Origene) using lipofectamine2000 for 48 h. For the establishment of a stable cell line overexpressing NLRX1 HEK293 cells were transfected with pCMV‐hNLRX1 plasmids (Origene) using lipofectamine2000 and then maintained with G418 (200 μg/ml, Calbiochem).

For induction of premature senescence, HEK293 cells were treated hydrogen peroxide (H_2_O_2_, 200 μM) for 2 h. H_2_O_2_ was purchased from Baker^™^. For inhibition of mTOR, MEFs were treated with rapamycin (100 nM) for 16 h. Rapamycin was purchased from MP Biomedicals.

### 4.4 Western blot analysis

Cells or tissues were lysed in lysis buffer containing protease/phosphatase inhibitors (GenDEPOT). The soluble fraction was separated by centrifugations at 13,000 × g for 10 min at 4°C. The concentration of total protein in the cleared lysate was measured using a BCA Assay kit (Thermo). The samples were resolved in SDS‐PAGE, and the detection was carried out using Pierce ECL Western blotting substrate (Thermo Fisher Scientific, Inc.). The blots were exposed to a ChemiDoc MP Imaging System (BioRad). The quantification of protein expression was carried out by optical densitometry and analyzed using the ImageJ software (ImageJ 1.53v, National Institutes of Health). Antibody information in detail is provided in Table S1.

### 4.5 IL‐6 and IL‐1β measurement

For the induction of IL‐6 and IL‐1β, MEFs were primed with 1 µg/ml LPS (Sigma) for 4 h, and then treated with 5 µM Nigericin (Invivogen) for 1 h. IL‐6 and IL‐1β were measured by ELISA assay using Mouse IL‐6 or IL‐1β DuoSet (R&D Systems) according to the manufacturer's protocol.

### 4.6 Real‐time PCR

Total RNA was extracted from the lung tissues using RNeasy kit (QIAGEN) and stored at −80°C until reverse transcription. Total RNA was quantified by quantitative PCR using Nanodrop (Life Technologies). The cDNA was analyzed by SYBR‐Green Supermix (Bio‐rad) with an ABI 7500 fast real‐time PCR (Applied Biosystems) and normalized to 18S expression. Data are expressed in relative fold change. Primer information is listed in Table S2.

### 4.7 Senescence‐associated beta‐galactosidase assay

Replicative senescence was induced by normal subculture. Senescent cells from WT and NLRX1 KO MEFs at passages 2, 4, and 6, respectively, were identified with the senescence‐associated β‐galactosidase kit (Cell Signaling) according to the manufacturer's protocol. The images of β‐Gal‐stained cells were captured by EVOS XL core cell imaging microscope (Life Technologies). Quantification of SA β‐gal‐positive cells was analyzed by ImageJ software (ImageJ 1.53v, National Institutes of Health). SPiDER‐β‐Gal (dojindo) and Hoechst (Invtrogen) were used to detect fluorescence of SA‐β‐Gal activity, and fluorescent images were captured using Leica TCS SP5 confocal microscope and analyzed with LAS X Life Science software.

### 4.8 Cell growth rate and proliferation assay

For each passage, the same numbers of MEFs were seeded on culture plates, harvested, and collected after 2 days for counting cell numbers. After the counting, the same numbers of MEFs were re‐seeded for the next passage. Cell growth rate per passage was calculated from the collected cell numbers divided by the seeded cell numbers. Cell proliferation was measured by 3‐(4,5‐dimethylthiazol‐2‐yl)‐2,5‐diphenyltetrazolium bromide (MTT) colorimetric method using CellTiter 96^®^ Non‐Radioactive Cell Proliferation Assay kit (Promega) according to the manufacturer's protocol.

### 4.9 Nicotinamide adenine dinucleotide/NADH measurements

Nicotinamide adenine dinucleotide/NADH Quantitation Colorimetric Kit (Biovision) was used following the manufacturer's protocol. The formula, “(NADtotal ‐NADH)/NADH,” was used for the calculation of the NAD^+^/NADH ratio.

### 4.10 H&E staining and histology

Left‐lung was inflated with 0.5% low temperature‐melting agarose at a constant pressure of 25 cm following the method as reported in our publication (Kang et al., 2008). The tissues were then fixed overnight with 10% neutral buffered formalin solution, embedded in paraffin, and sectioned for staining with hematoxylin and eosin (H&E). H&E‐stained sections were used to evaluate lung structure using upright microscope ECLIPSE Ni and analyzed by NIS‐Elements BR software. Mean chord lengths were measured by ImageJ software following the method as reported in our publication (Kang et al., 2015).

### 4.11 Pulmonary function test

Pulmonary function tests were performed following the manufacturer's protocol (flexiVent, SCIREQ). In brief, mice were anesthetized with an intraperitoneal injection of ketamine solution including xylazine. After tracheostomy, the needle was inserted into the trachea and fixed with a thread. The small animal ventilator (flexiVent, SCIREQ) and the intubated needle were connected and ventilated at a respiratory rate of 150 breaths/min. To prevent muscle tension, intraperitoneal injection of pancuronium bromide, a typical non‐depolarizing curare‐mimetic muscle relaxant at a concentration of 0.8 mg/kg, was given. Parameters measurements were performed using the single‐FOT maneuver (“Snapshot‐150 perturbation”) and the broadband FOT maneuver (“Quick Prime‐3 perturbation”). The median of at least three acceptable measurements was then calculated.

### 4.12 Statistical analysis

All statistical analyses were executed using the Prism (GraphPad, version 9) software program. Comparisons between two groups were performed with two‐tailed t test (unpaired). For the multiple comparisons, 1‐way or 2‐way ANOVA was used. Values are expressed as mean ± SEM or SD. Statistical significance was defined at a value of *p* less than 0.05.

## CONFLICT OF INTEREST

The authors declare no competing financial interests.

## AUTHOR CONTRIBUTIONS

HJS, SHK, and MJK conceived the idea and designed the experiments. HJS, SHK, and HJP performed the experiments. MSS and IK provided the important reagents/tools. HJS, IK, and MJK provided the scientific insight. HJS and MJK analyzed the data. HJS and MJK drafted the manuscript. All of the authors reviewed the manuscript.

## Supporting information

Fig S1‐S6

Supplementary Material

## Data Availability

The data that supports the findings of this study are available in the supplementary material of this article.
