# Supplementary material for: Nucleotide‐binding domain and leucine‐rich‐repeat‐containing protein X1 deficiency induces nicotinamide adenine dinucleotide decline, mechanistic target of rapamycin activation, and cellular senescence and accelerates aging lung‐like changes
Source: Aging Cell. 2021 Jun 4;20(7):e13410. doi: 10.1111/acel.13410 (PMC8282248; doi:10.1111/acel.13410)
Supplement: Supplementary file 2 — Supplementary Material [file ACEL-20-e13410-s001.docx]

**SUPPLEMENTAL INFORMATION**

NLRX1 deficiency induces NAD^+^ decline, mTOR activation and cellular senescence and accelerates aging lung-like changes

**Hyeon Jun Shin^1^, Sang-Hun Kim^1^, Hong-Jai Park^2^, Min-Sun Shin^2^, Insoo Kang^2^, and Min-Jong Kang^1,^** ^†^

**Material List**

**Fig S1.** Age-dependent decrease of NLRX1 in lungs and accelerated aging lung-like structural changes in NLRX1 deficiency *in vivo*.

**Fig S2.** Age-dependent accelerated aging lung-like functional changes in NLRX1 deficiency *in vivo*.

**Fig S3.** mTOR signaling not significantly altered to NLRX1 deficiency in 1- or 2-month-old mice lung.

**Supplemental Table 1.** Antibodies used in this study for Western blot

**Supplemental Table 2.** Primers used in this study for Real-time PCR

Separate file

**Supplemental Data 1.** Raw Data

**Fig S1. Age-dependent decrease of NLRX1 in lungs and accelerated aging lung-like structural changes in NLRX1 deficiency *in vivo*.** (**a**) Immunoblot to detect the expression level of NLRX1 in 3-, 12-, or 24-month-old (mo) lungs from wild-type (WT) mice. (**c and d**) Representative histology images (H&E staining) of 3- or 24-mo lung sections from WT or NLRX1 knockout (KO) mice. Scale bars, 200 μm (**b**), Scale bars, 400 μm (**c**).

**Fig S2. Age-dependent accelerated aging lung-like functional changes in NLRX1 deficiency *in vivo*.**

The results of pulmonary function tests from 3-, 6-, 16- or 24-month-old (mo) wild type (WT) or NLRX1 knockout (KO) mice measured by flexiVent system. WT or NLRX1 KO 3-mo, n = 7; WT or NLRX1 KO 6-mo, n = 6; WT or NLRX1 KO 16-mo, n = 8; WT 24-mo, n=7; NLRX1 KO 24-mo, n=4. * Note: Two of six NLRX1 KO mice allocated for the 24-mo group succumbed to natural death during the follow-up period. Graphs showing (**a**) inspiratory capacity (IC) of lungs from 3-, 6-, 16- or 24-mo WT or KO mice; (**b**) dynamic compliance of respiratory system (Crs); (**c**) static compliance of respiratory system (Cst); (**d**) elastance of respiratory system (Ers); (**e**) tissue elastance (H); (**f**) tissue damping (G). Error bars indicate means ± SEM. Data were analyzed by two-way ANOVA followed by Tukey’s multiple comparisons test. ^#^*P* < 0.001, ***P* < 0.01, **P* < 0.05.

**Fig S3. mTOR signaling not significantly altered to NLRX1 deficiency in 1- or 2-month-old mice lung.** (**a**) Immunoblot to detect NLRX1, mTOR, phosphorylation of mTOR at serine 2448 (p-mTOR^S2448^) and 4EBP1 in lungs from 1- or 2-month-old (mo) wild-type (WT) or NLRX1 knockout (KO) mice, respectively. (**b**) Graph represents relative expression levels of mTOR or p-mTOR^S2448^ (n = 3 mice, each group). Error bars indicate means ± SEM. Data were analyzed by two-way ANOVA followed by Tukey’s multiple comparisons test. ^#^*P* < 0.001, ***P* < 0.01, **P* < 0.05. N.S. means not significant.

**Supplemental Table 1. Antibodies used in this study for Western blot**

| **Antibodies** | **Source** | **Identifier** |
| --- | --- | --- |
| Rabbit monoclonal anti-NLRX1 (D4M3Z) | Cell signaling technology | Cat# 13829 |
| Rabbit monoclonal anti-p53 (D2H9O) (Rodent Specific) |  | Cat# 32532 |
| Rabbit monoclonal anti-mTOR (7C10) |  | Cat# 2983 |
| Rabbit monoclonal anti-phospho-mTOR (Ser2448) (D9C2) |  | Cat# 5536 |
| Rabbit monoclonal anti-CDKN2A/p16INK4a | Abcam | Cat# ab211542 |
| Mouse monoclonal anti-SIRT1 |  | Cat# ab110304 |
| Mouse monoclonal anti-Total OXPHOS cocktail (Rodent specific) |  | Cat# ab110413 |
| Rabbit polyclonal anti-phospho-p53 (Ser15) |  | Cat# ab1431 |
| Mouse monoclonal anti-β-Actin (HRP Conjugate) | Santa Cruz Biotechnology | Cat# sc-47778 |

**Supplemental Table 2. Primers used in this study for Real-time PCR**

| **Gene** | **Forward primer (5’→3’)** | **Reverse primer (5’→3’)** |
| --- | --- | --- |
| *Nlrx1* | TAGGGCCTTTATCCGTTACCA | TAAACCACTCGGTGAGGTTCC |
| *18s* | GTAACCCGTTGAACCCCATT | CCATCCAATCGGTAGTAGCG |
